# Effects of disinfectant wipes on sensitive healthcare surfaces

**DOI:** 10.1186/2047-2994-4-S1-P40

**Published:** 2015-06-16

**Authors:** D Del Re, C Ikeno, K Smid, D Swift

**Affiliations:** 1 Biolennia Laboratories, Toronto, Canada; 2 Micrylium Laboratories, Toronto, Canada; 3 BDL, Canada

## Introduction

Studies have shown that shared surfaces and devices can serve as a route for transmission of pathogens; however, proper disinfection protocols are lacking to address sensitive surfaces and equipment that can be permanently damaged by disinfectants used in healthcare environments.

## Objectives

The main objectives of this study were to test commercially available disinfectant wipes on sensitive surfaces to examine antimicrobial efficacy as well as any damaging effects.

## Methods

Samples were taken before and after use of a disinfectant wipe from touch screens, keyboards, and computer mice at 4 Long Term Care (LTC) facilities across the Greater Toronto Area. Pieces of mattress coverlet and touch screens were wiped approximately every hour for two months with various disinfectant wipes and examined for any damage.

## Results

All surfaces sampled at LTC facilities showed marked contamination with bacteria and fungi prior to disinfection. After wiping with Product T, samples were cleared of contamination.

Touch screens and mattress coverlets showed no damage after repeated wiping with Products S and T. Discoloration and damage were observed with Products C, V, and P. Some surfaces showed contamination with *S. aureus* and *E. coli*. Variable results were observed for antimicrobial effectiveness; some wipes showed complete removal of organisms while others showed some to no reduction.

## Conclusion

This study further illustrates that shared surfaces and devices can be contaminated with microorganisms, stressing the importance of disinfection of these surfaces. Sensitive surfaces present a challenge to disinfection; however, we have demonstrated that products are available to effectively disinfect sensitive surfaces without causing harmful and costly damage.

## Disclosure of interest

None declared.

